# Implementation of innovative medical technologies in German inpatient care: patterns of utilization and evidence development

**DOI:** 10.1186/s13012-021-01159-3

**Published:** 2021-10-30

**Authors:** Marie Dreger, Helene Eckhardt, Susanne Felgner, Hanna Ermann, Hendrikje Lantzsch, Tanja Rombey, Reinhard Busse, Cornelia Henschke, Dimitra Panteli

**Affiliations:** 1 Department of Health Care Management, Technische Universität Berlin, Straße des 17. Juni 135, 10623 Berlin, Germany; 2 Berlin Centre for Health Economics Research (BerlinHECOR), Technische Universität Berlin, Straße des 17. Juni 135, 10623 Berlin, Germany

**Keywords:** Adoption, Implementation, Diffusion patterns, Evidence, Clinical trials, Value-based health care, Medical technologies, Inpatient care, Germany

## Abstract

**Background:**

Innovative medical technologies are commonly associated with positive expectations. At the time of their introduction into care, there is often little evidence available regarding their benefits and harms. Accordingly, some innovative medical technologies with a lack of evidence are used widely until or even though findings of adverse events emerge, while others with study results supporting their safety and effectiveness remain underused. This study aims at examining the diffusion patterns of innovative medical technologies in German inpatient care between 2005 and 2017 while simultaneously considering evidence development.

**Methods:**

Based on a qualitatively derived typology and a quantitative clustering of the adoption curves, a representative sample of 21 technologies was selected for further evaluation. Published scientific evidence on efficacy/effectiveness and safety of the technologies was identified and extracted in a systematic approach. Derived from a two-dimensional classification according to the degree of utilization and availability of supportive evidence, the diffusion patterns were then assigned to the categories “Success” (widespread/positive), “Hazard” (widespread/negative), “Overadoption” (widespread/limited or none), “Underadoption” (cautious/positive), “Vigilance” (cautious/negative), and “Prudence” (cautious/limited or none).

**Results:**

Overall, we found limited evidence on the examined technologies regarding both the quantity and quality of published randomized controlled trials. Thus, the categories “Prudence” and “Overadoption” together account for nearly three-quarters of the years evaluated, followed by “Success” with 17%. Even when evidence is available, the transfer of knowledge into practice appears to be inhibited.

**Conclusions:**

The successful implementation of safe and effective innovative medical technologies into practice requires substantial further efforts by policymakers to strengthen systematic knowledge generation and translation. Creating an environment that encourages the conduct of rigorous studies, promotes knowledge translation, and rewards innovative medical technologies according to their added value is a prerequisite for the diffusion of valuable health care.

**Supplementary Information:**

The online version contains supplementary material available at 10.1186/s13012-021-01159-3.

Contributions to the literature

- This study presents a novel methodological approach for categorizing utilization patterns based on hospital data and selecting a balanced sample of technologies for analysis.
- It analyzes the relationship between utilization and scientific evidence across a comprehensive sample of 21 technologies; this has previously mostly been studied for individual technologies.
- Several of the 21 technologies were overadopted (utilized widely despite limited or no evidence), which entails risks for patients and health systems.
- Our findings reinforce the need for reconsidering how innovation systems work: how evidence is generated and disseminated, and how it is embedded in (de)implementation decisions in policy and practice.

## Background

Innovative medical technologies are commonly accompanied by positive expectations. In fact, they may reduce pain and/or contribute to patients’ full recovery, improve health-related quality of life and well-being, and even create economic benefits by reducing health care expenditures in the long run [1]. As a result, an initially uncritical adoption behavior often prevails [2]. However, it is not uncommon for these high expectations to prove unrealizable. This not only puts patients at risk of suffering health damages, it can also mean unwarranted high expenses for health systems [3].

The World Health Organization (WHO) defines health technology as “any application of organized knowledge and skills in the form of medicines, medical devices, vaccines, procedures and systems, developed to solve a health problem and improve quality of life” [4]. Many innovative medical technologies—both therapeutic and diagnostic—are procedures, in which medical devices constitute a central component. Historically, medical devices in the European Union (EU) have had to undergo a conformity assessment demonstrating that they fulfill regulatory requirements in terms of quality, safety, and performance in order to obtain the Conformité Européenne (CE) mark, which certifies their approval for marketing. In other countries, such as the USA, an additional demonstration of efficacy may be required depending on the risk associated with the device [5]. There are several documented examples of devices approved for market in the EU and not in the USA, which resulted in major patient harm or were later found to be ineffective. For example, the drug-eluting stent CoSTAR had already been licensed in the EU when an approval trial in the USA showed increased rates of reinterventions and heart attacks [6]. Despite the increased rigor of the marketing authorization process introduced by the medical device regulation (Regulation (EU) 2017/245), the requirements for pre-market evaluation of medical devices remain less strict than those for pharmaceuticals [7], although the potential for harm is not necessarily lower.

In many countries, a positive (cost-)effectiveness evaluation is required before new medical technologies can be reimbursed in publicly funded health systems [8]. Usually, this is not the case in German inpatient care. New technologies may be used unless they are explicitly excluded by the Federal Joint Committee (*Gemeinsamer Bundesausschuss*, G-BA), the highest decision-making body in the German health care system [9]. If new technologies cannot yet be appropriately reimbursed via per-case flat rates and additional charges of the German-diagnosis related groups (G-DRG) system, extrabudgetary innovation payments may apply. However, to receive permission to enter into negotiations with health insurers for such payments, hospitals must go through an application process for each technology they want to use (see Henschke et al. [10] for details on this process). The negotiated amount does not depend on the technology’s efficacy/effectiveness or risk-benefit profile. In 2016, the concept of the innovation payments was supplemented by a structured obligatory early benefit assessment for certain medical technologies based on high-risk medical devices, so-called new diagnostic and treatment methods (*Neue Untersuchungs- und Behandlungsmethoden*, NUB); but this pathway has rarely been activated [11]. The decision to use these new medical technologies, and consequently their diffusion, is largely the responsibility of hospitals. Their implementation is not necessarily based on evidence-based recommendations [12]. It happens that some innovative medical technologies are utilized widely despite a lack of supporting evidence while others with findings supporting their effectiveness and safety remain underused [13, 14].

Research on diffusion of innovation was largely shaped by Rogers [15], who argued that diffusion is facilitated by the perceived relative advantage of new technologies; their compatibility with previous experience, values, and needs; their subjective complexity; and their trialability [15, 16]. However, the perceived relative advantage to current practice appears to be the subjective result of debate among health professionals and not a rational assessment based on credible evidence [13, 17]. Indeed, previous research demonstrates that adoption and diffusion of innovations depend only partially on scientific evidence [18] and are shaped by many intra- and extra-organizational factors [19, 20], not least environmental factors such as financial incentives [21].

Because the adoption and diffusion of innovative technologies are important drivers of value-based health care [14], it is important to understand the optimal timing for them to be (de)adopted, particularly in relation to the availability of robust scientific evidence. Considering the fast pace of innovation in health care and the resulting broad range of new technologies, it is also important to recognize that diffusion patterns will differ, and that research on this relationship should strive to capture this variability. Therefore, this study had two aims:

- To develop a methodological approach for categorizing adoption curves of innovative medical technologies based on hospital utilization data and available scientific evidence, in order to select a varied sample for further investigation.
- To investigate how the diffusion patterns of the selected technologies are associated with the underlying scientific evidence on safety and efficacy/effectiveness in the German health care system, which is characterized by low entry requirements for utilization in inpatient care.

## Methods

**Fig. 1:**
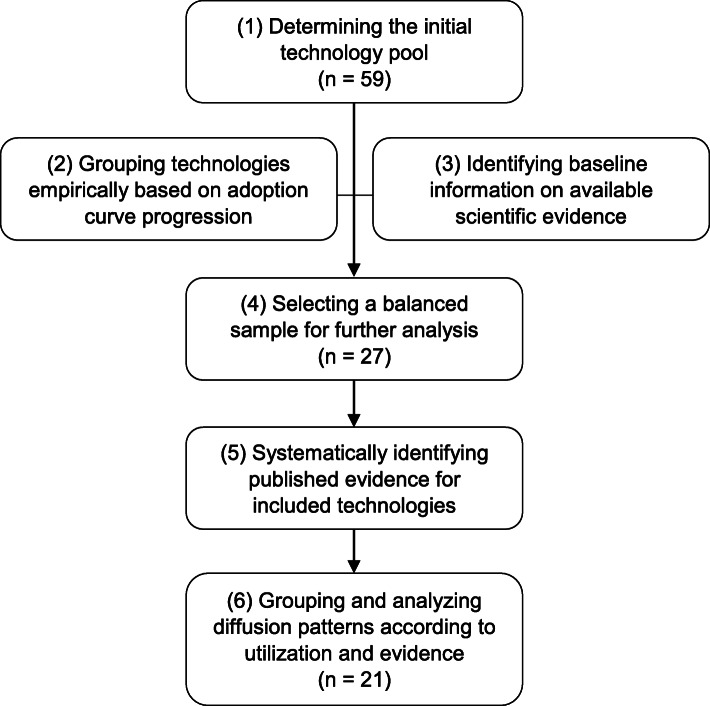
Methodology flowchart

Innovative medical technologies reimbursed in German inpatient care between 2005 and 2017 were identified on the basis of the lists published annually by the German Institute for the Hospital Remuneration System (*Institut für das Entgeltsystem im Krankenhaus*, InEK) [22]. Those with a relevant number of cases were pre-selected for consideration. Data on utilization between 2005 and 2017 were drawn from the German DRG statistics [23] and used to plot adoption and diffusion curves and determine the number of cases treated with appropriate alternatives. The adoption curves were grouped in seven progression types. A minimum number of technologies per type were selected for investigation, also considering available evidence. The maximum sample for investigation was set at 30 technologies to balance representativity with feasibility. The diffusion curves were subsequently juxtaposed to systematically identified randomized controlled trials (RCTs) over the observation period. Figure 1 visualizes the methodical approach in a flowchart. Each step is described in detail below. Since steps 1 to 5 serve as preparation for the analysis in step 6, their outcomes are described here and not separately in the “Results” section.

### Step 1: Determining the initial technology pool

To select new technologies for the study, the following actions were taken:

i) The lists of new medical diagnostic and treatment methods published annually by the InEK between 2005 and 2012 were scrutinized. These lists include all new medical diagnostic and treatment methods for which hospitals have requested permission to negotiate innovation payments with health insurers (as they are not adequately reimbursed by current DRGs). The time window was chosen to ensure that data was available for at least five years prior to the start of this research. Consequently, the observation period for this study spans the years 2005 to 2017.
ii) The “DRG statistics” dataset, which includes hospital claims data reported annually to the German Federal Office of Statistics, was used to determine the number of hospitals implementing the technology and the total number of cases. DRG statistics capture anonymized information for all inpatient treatments.
iii) The following criteria were applied for selection based on the information from (i) and (ii):

- Permission to negotiate an innovation payment for the technology was requested by more than ten hospitals and granted for at least 1 year between 2005 and 2012
- Case numbers and numbers of hospitals implementing the technology were available for at least 4 years
- 100 cases or more were reimbursed for at least 1 year

Overall, 59 technologies were included following these selection criteria and are listed in Table 1 (Table 1 also shows the final selection of 27 technologies based on the process described in step 4, below). They can be classified into ten different anatomical regions of application. More than two-thirds of the technologies (41 of 59) concern the cardiovascular system.

**Table 1:** Included innovative technologies

Type	Technology	Abbreviation	Selection via adoption curve progression type no. or evidence cross-check
**Procedures on nervous system**			
	Neurostimulator for stimulation of the spinal cord or peripheral nervous system, rechargeable	NEURO	
	Baroreceptor activation	BRA	
**Procedures on ear and mastoid process**			
	Hybrid cochlear implant	HCI	
**Procedures on respiratory system**			
	Lung volume reduction by insertion of coils	LVRC	4
	Endobronchial valve	EBV	
	Pumpless extracorporal lung assist/interventional lung assist	PECLA/iLA	Evidence cross-check
**Procedures on cardiovascular system**			
	Percutaneous transluminal clipping for mitral valve regurgitation	MR-PTC	
	Transcatheter aortic valve implantation	TAVI	1
	Minimally invasive heart valve surgery (endovascular implantation of pulmonary valve replacement)	PVR	
	Mitral valve annuloplasty with clamp	MVAC	5
	Minimally invasive operations on heart valves (implantation of a mitral valve replacement)	MVR	
	Percutaneous ventricular assist device (microaxial blood pump)	pVAD	6.1
	Excimer laser extraction of pacemaker and defibrillator electrodes	EL-P/ICD	3
	Defibrillator with subcutaneously implantable electrode	S-ICD	
	Cardiac event recorder after ablative measures for atrial fibrillation/atrial tachycardia	ER-ABL	6.2
	Coronary bifurcation stents	CBS	
	Antibody coated coronary stent	ACCS	
	Bioresorbable vascular scaffold in coronary vessels	BVS	4
	Self-expanding bare metal stents in coronary vessels	SE-BMS	2
	Coronary stent, self-expanding (at least two stents, drug-eluting)	SE-DES	
	Drug-coated balloon catheter in coronary vessels	DCB-CV	3
	Drug-coated balloon catheter in intracranial vessels	DCB-IV	6.2
	Extra-long coils (3D) for intracranial aneurysm therapy	IAELC	
	Volume coils for intracranial aneurysm therapy	IAVC	
	Intraaneurysmal hemodynamically effective implant for endovascular treatment of intracranial aneurysms.	IAHEI	
	Bioactive coils for intracranial aneurysm therapy	IABC	Evidence cross-check
	Bioactive extra-long coils for intracranial aneurysm therapy	IABC-EL	
	Flow-diverter (hemodynamically effective implant for endovascular treatment) in intracranial vessels	FD-IV	
	Intracranial endovascular thrombectomy (microwire retriever)	IET-MICRO	6.2
	Drug-coated balloon catheter in visceral vessels	DCB-VV	
	Insertion of coated (covered) stents with bioactive surface for visceral and supraaortic vessels	BS-VSAV	
	Drug-eluting stents for the treatment of lesions of the supraaortic arteries	DES-SAA	
	Drug-coated balloon catheter in thoracic vessels	DCB-TV	
	Fenestrated endoprostheses for abdominal aortic aneurysms	FE-AAA	
	Drug-coated balloon catheter in abdominal vessels	DCB-AV	1
	Insertion of coated (covered) stents with bioactive surface for peripheral vessels	BS-PV	
	Drug-coated balloon catheter in shoulder and upper arm vessels	DCB-SUAV	
	Drug-coated balloon catheter in lower arm vessels	DCB-LAV	
	Flow-diverter (hemodynamically effective implant for endovascular treatment of peripheral aneurysms) in upper leg vessels	FD-ULV	5
	Drug-coated balloon catheter in upper leg vessels	DCB-ULV	1
	Drug-coated balloon catheter in lower leg vessels	DCB-LLV	1
	Implantation of a drug-eluting stent in lower leg vessels	DES-LLV	6.1
	Implantation of a drug-eluting stent in upper leg vessels	DES-ULV	6.1
	Drug-coated balloon catheter in artificial vessels	DCB-ARTV	
	Drug-coated balloon catheter in other vessels	DCB-OTHV	
	Endovascular implantation/repair of a stent prosthesis using an endostapler	SP-ENDOST	
	Endoaortic balloon occlusion with extracorporeal circulation	EABO	Evidence cross-check
**Procedures on digestive system**			
	Esophageal sphincter implant, magnetic	MESI	
**Procedures on urinary system**			
	Adjustable continence therapy	ACT	4
	Double J metal stent for urinary diversion in ureteral strictures	UD-DJMS	
	Fluorescence-assisted transurethral resection	F-TUR	6.2
	Anticoagulation with citrate during dialysis	ACD	6.1
	Dialysis with high cut-off dialysis membrane	HCO	Evidence cross-check
**Obstetric procedures**			
	Fetoscopic drainage therapy	FDT	Evidence cross-check
**Procedures on musculoskeletal system**			
	Vertical expandable prosthetic titanium rib	VEPTR	
	Therapy of scoliosis by means of magnetic-controlled rods	SCO-MAGN	
**Antineoplastic procedures**			
	Drug-eluting beads for transarterial chemoembolization	DEB-TACE	4
**Diagnostic procedures**			
	Ex vivo chemosensitivity testing	EVCT	
	Molecular monitoring of residual tumor burden	MRD	3

### Step 2: Grouping technologies empirically based on adoption curve progression

Based on the DRG case data described above, adoption curves were plotted for all 59 technologies (visualized in Additional file 1). We subsequently empirically developed seven curve progression types in order to select a varied sample for further analysis. The types were developed by grouping curve progressions that were as homogeneous as possible; this was achieved in two stages.

First, qualitative clustering was performed with the aim of unambiguous assignment of the curve progressions. The gradients of the curves over time, changes in the gradient, and the approach of the curve to a saturation point were considered. The operationalization of these criteria and the resulting groups can be traced in Table 2 (types I-V). According to this approach, 28 of the 59 technologies could not be assigned to any of the five types. These were initially grouped together in a further group (VI) under the keyword “complex.”

**Table 2:** Types of adoption curves and associated technologies

Type	Criterion	Technologies (no.)
(I) Continuous increase	$\frac{\mathrm{d}f(t)}{\mathrm{d}t}=: m\ (t)>0$ ∀ *t* ∈ [2006; 2017]	HCI, MR-PTC, S-ICD, IAHEI, SCO-MAGN, BS-PV, SE-DES, TAVI, BS-VSAV, DCB-SUAV, DCB-LAV, DCB-AV, SCB-ULV, DCB-LLV, DCB-ARTV (*n* = 15)
(II) Continuous decrease	$\frac{\mathrm{d}f(t)}{\mathrm{d}t}=: m\ (t)<0$ ∀ *t* ∈ [2006; 2017]	SE-BMS (*n* = 1)
(III) Reaching a saturation	$\frac{f(2015)}{f(2014)}<1.1\,\wedge$ $\frac{f(2016)}{f(2015)}<1.075\,\wedge$ $\frac{f(2017)}{f(2016)}<1.05$ *f*(2015) > *f*(*t*) ∀ *t* < 2015	EL-P/ICD, MRD, DCB-CV (*n* = 3)
(IV) “Local maximum”: Continuous increase followed by continuous decrease	*m*(*t*_*I*_) > 0 ∧ *m*(*t*_II_) < 0 *t*_I_ ∈ [*t*_*i*_; *t*_*j*_], *t*_II_ ∈ [*t*_*j*_; *t*_*k*_] *t*_*i*_, *t*_*j*_ < *t*_*k*_	FE-AAA, ACT, ACCS, DEB-TACE, LVRC, BVS, BRA, IABC, MVR, FD-IV (*n* = 10)
(V) “Local minimum”: Continuous decrease followed by continuous increase	*m*(*t*_*I*_) < 0 ∧ *m*(*t*_II_) > 0 *t*_I_ ∈ [*t*_*i*_; *t*_*j*_], *t*_II_ ∈ [*t*_*j*_; *t*_*k*_] *t*_*i*_, *t*_*j*_ < *t*_*k*_	MVAC, FD-ULV (*n* = 2)
(VI) Complex	NA	
(VI.a)	According to hierarchical-agglomerative clustering method (see Additional file 2)	PECLA/iLA, pVAD, EVCT, CBS, ACD, VEPTR, IAELC, UD-DJMS, DES-LLV, EABO, HCO, IAVC, SP-ENDOST, FDT, DES-SAA, MESI, EBV, NEURO, IABC-EL, DES-ULV, PVR, DCB-TV, DCB-OTHV (*n* = 23)
(VI.b)	According to hierarchical-agglomerative clustering method (see Additional file 2)	IET-MICRO, F-TUR, DCB-IV, DCB-VV, ER-ABL (*n* = 5)

To obtain further differentiation of this residual group of curve progressions, a quantitative cluster analysis [24] was applied in a second stage using the statistical software RStudio (Version 1.3.1093). Details are presented in detail in Additional file 2. Based on the resulting target number of two clusters, the 28 technologies in curve progression type VI were distributed into two groups of 23 (type VI.a) and 5 technologies (type VI.b), respectively. Table 2 summarizes the seven types of progression curves, the operationalization of the criteria, and the distribution of technologies to the types.

### Step 3: Identifying baseline information on available scientific evidence

The aim of this step was to gather baseline information on the availability of evidence on the safety and efficacy/effectiveness for each technology to ensure that the selected sample covers different types of adoption curves as well as different results derived from the available evidence (i.e., evidence supporting utilization with or without restrictions, evidence not supporting utilization). For this purpose, we screened reports from the Medical Review Board of the Federal Association of Sickness Funds (*Medizinischer Dienst des Spitzenverbandes Bund der Krankenkassen*, MDS) for the 59 technologies described above. These reports are prepared upon request to evaluate technologies for which the negotiation of innovation payments has been permitted by the InEK.

Since the reports are confidential, detailed results cannot be discussed in this paper. A total of 45 reports were identified for 56 of the 59 included technologies. We developed a structured template to extract information on methodology (e.g., year, PICOS criteria), included studies (e.g., level of evidence), reported outcomes (mortality, morbidity, quality of life), results of included studies, and the conclusions of the MDS appraisal. Based on the latter, we classified each technology into one of the evidence groups “has potential without limitations” (1), “has potential for certain patients” (2), and “no potential” (3). It is important to note that these initial assessments were not necessarily identical to the results of the systematic identification of evidence described in step 5, below.

### Step 4: Selecting a balanced sample for further analysis

Of the 59 technologies pre-selected in step 1, we had to select a maximum of 30 technologies for feasibility reasons, containing all curve progression types and evidence classifications. Within these groups, we chose those technologies that were most relevant to care based on high case numbers and high rates of changes in case numbers. The conjunction of these two parameters was translated into a multiplicative criterion for each technology *i* from all available data years *j*:

$$Crit{.}_i=\left(\mathrm{Maximum}\ \mathrm{number}\ \mathrm{of}\ {\mathrm{cases}}_i\right)\bullet \left(\mathrm{Largest}\ \mathrm{rate}\ \mathrm{of}\ {\mathrm{change}}_i\right)=\underset{j\in \left[1,13\right]}{\max}\left\{{f}_i\left({t}_j\right)\right\}\bullet \underset{j\in \left[1,13\right]}{\max}\left\{\left|\frac{f_i\left({t}_{j+1}\right)}{f_i\left({t}_j\right)}-1\right|\right\}$$

Within each type of curve progression, all technologies were sorted by size based on *Crit*._*i*_ in descending order. To achieve a sample of 28 (as a multiple of the seven groups), the four top technologies were selected from each group (i.e., I-V, VI.a, and VI.b). However, since curve types II, III, and V each contain fewer than four technologies, the resulting sample included only 22. To further diversify the sample and balance the selection of evidence classifications, the remaining technologies were filtered according to the following approach.

First, technologies used in the context of an indication already strongly represented in the sample of 22 were excluded. Then, three additional technologies were selected from the evidence group “has potential without limitations” and two from the group “no potential”, as these were the least represented. This resulted in almost even distribution across the evidence groups: 55% (6 of 11) selected from group 1 (“has potential without limitations”), 60% (3 of 5) selected from group 2 (“has potential for certain patients”), and 57% (4 of 7) selected from group 3 (“no potential”). This sample also achieved a balance between groups with clear (13 of 27) and unclear evidence (14 of 27). The selection of these five additional technologies was also based on curve progressions. Candidates were compared qualitatively and those that appeared to be particularly complementary to the technologies already in the selected sample were included. We thus aimed to obtain the broadest possible spectrum of examined technologies. The final sample of 27 is shown in the last column of Table 1.

### Step 5: Systematically identifying published evidence for included technologies

Subsequently, published evidence on the selected technologies was systematically identified, selected, and evaluated. For this purpose, bibliographic biomedical electronic databases (Medline and Embase via OVID, PubMed, the Cochrane Library), clinical trial registries (Clinicaltrials.org, WHO International Clinical Trials Registry Platform (ICTRP)), and selected Health Technology Assessment (HTA) databases and agencies (LBI-HTA, IQWiG/G-BA, CRD HTA/ INAHTA Database, DIMDI-DAHTA, EUnetHTA) were searched between May and September 2019. Search strategies with high sensitivity were used, and no restriction on the study designs was included. However, only RCTs are considered for the purpose of this article; an overview of all evidence types is presented elsewhere [25].

The results of the searches were imported into the literature management program EndNote (version x9, Clarivate). Explicit inclusion and exclusion criteria were formulated for each technology; inter alia, studies were included if they were published in the 2-year period before the first documented hospital case through the end of the observation period in 2017. Due to the number of included technologies and the high number of hits resulting from the sensitive searches, a rapid review approach [26] was adopted for selection of relevant citations. After duplicate removal, a random sample of 10% of all hits (at least 100) was drawn for each technology, and a title/abstract screening was carried out by two researchers independently. In case of discrepancies, the inclusion and exclusion criteria were discussed and adjusted, involving a third researcher if necessary. Subsequently, the remaining hits per technology were screened by one person (title/abstract screening followed by full-text screening as per standard systematic review methodology). Data from included publications were extracted using a standardized extraction sheet.

Based on the aggregated assessment of all outcomes by the authors in the “Conclusions” section, each publication was labeled based on its key message:

- Positive: The authors’ conclusions are consistently positive regarding efficacy/effectiveness and safety, and across patient groups. When a neutral (e.g., “equally safe”) and a positive (e.g., “effective”) statement were combined, the publication was considered positive.
- Negative: The authors’ conclusions are consistently negative regarding efficacy/effectiveness and safety, and across patient groups. When a neutral (e.g., “safe”) and a negative (e.g., “less efficacious”) statement were combined, the publication was classified as negative.
- Neutral: The authors conclude no difference between intervention and comparative intervention.
- Inconclusive: The authors conclude that no definitive statement can be made.

### Step 6: Grouping and analyzing diffusion patterns according to utilization and evidence

**Fig. 2:**
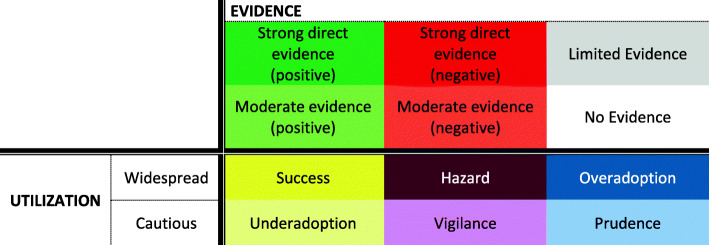
Six-field table for the classification of diffusion patterns based on utilization and evidence. Source: Authors’ own, adapted from Denis et al. [12]

In a final step, the diffusion of innovative medical technologies into the German health care system was examined against the background of available scientific evidence in order to identify successful and failed diffusion patterns and possible changes therein. For this purpose, we adapted the grid design for classification of health care innovations from Denis et al. [13]. The expressions of available scientific evidence and utilization are combined in a six-field table (see Fig. 2). In the case of positive scientific evidence, widespread utilization is described as “Success,” whereas cautious utilization implies “Underadoption.” We extended the initial matrix by Denis et al. [13] to include the case of negative scientific evidence, where widespread utilization results in “Hazard” and cautious utilization represents “Vigilance.” Limited or no available evidence may indicate “Overadoption” or, on the contrary, “Prudence.”

**Fig. 3:**
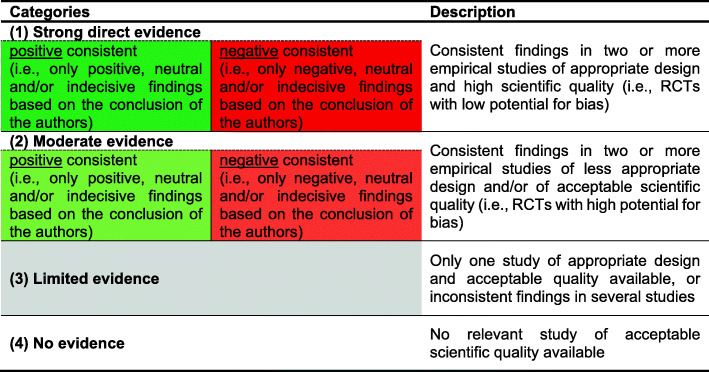
Categorization of evidence. Source: Authors’ own, adapted from Øvretveit [25] and Greenhalgh et al. [26]

The identified body of *"Evidence"* for each technology was categorized using a modified version of the World Health Organization/Health Evidence Network criteria [27, 28] (see Fig. 3). The three possible categories include strong direct or moderate evidence with positive conclusions, strong direct or moderate evidence with negative conclusions, and limited or no evidence. We categorized the evidence for each year and each technology based on identified RCTs (see Fig. 3). Included RCTs were assessed regarding risk of bias according to the procedural rules of the G-BA [29]; these bare resemblance to other relevant approaches, such as the Cochrane Risk of Bias tool. A high potential for bias led to the RCT in question being considered moderate rather than strong evidence.

*“Utilization”*, on the other axis of the classification grid in Fig. 2, is divided according to the diffusion rate in percent based on Roger’s diffusion of innovation model [15]. Accordingly, we set a threshold at 16% of the target population for each technology. Below this threshold, adoption of the technology is considered “cautious,” or commensurate with the risk of a novel technology without scientific support. Above the threshold, utilization of the technology was classified as widespread. It is important to note that Rogers’ model uses health care providers as the unit of analysis, with the first 16% corresponding to the group of innovators and early adopters, and above that to the (early) majority. We considered this threshold to be transferrable for classifying diffusion patterns based on case numbers, which offer an overarching view of technology diffusion. To our knowledge, there are no other models for such an exercise in the literature.

**Fig. 4:**
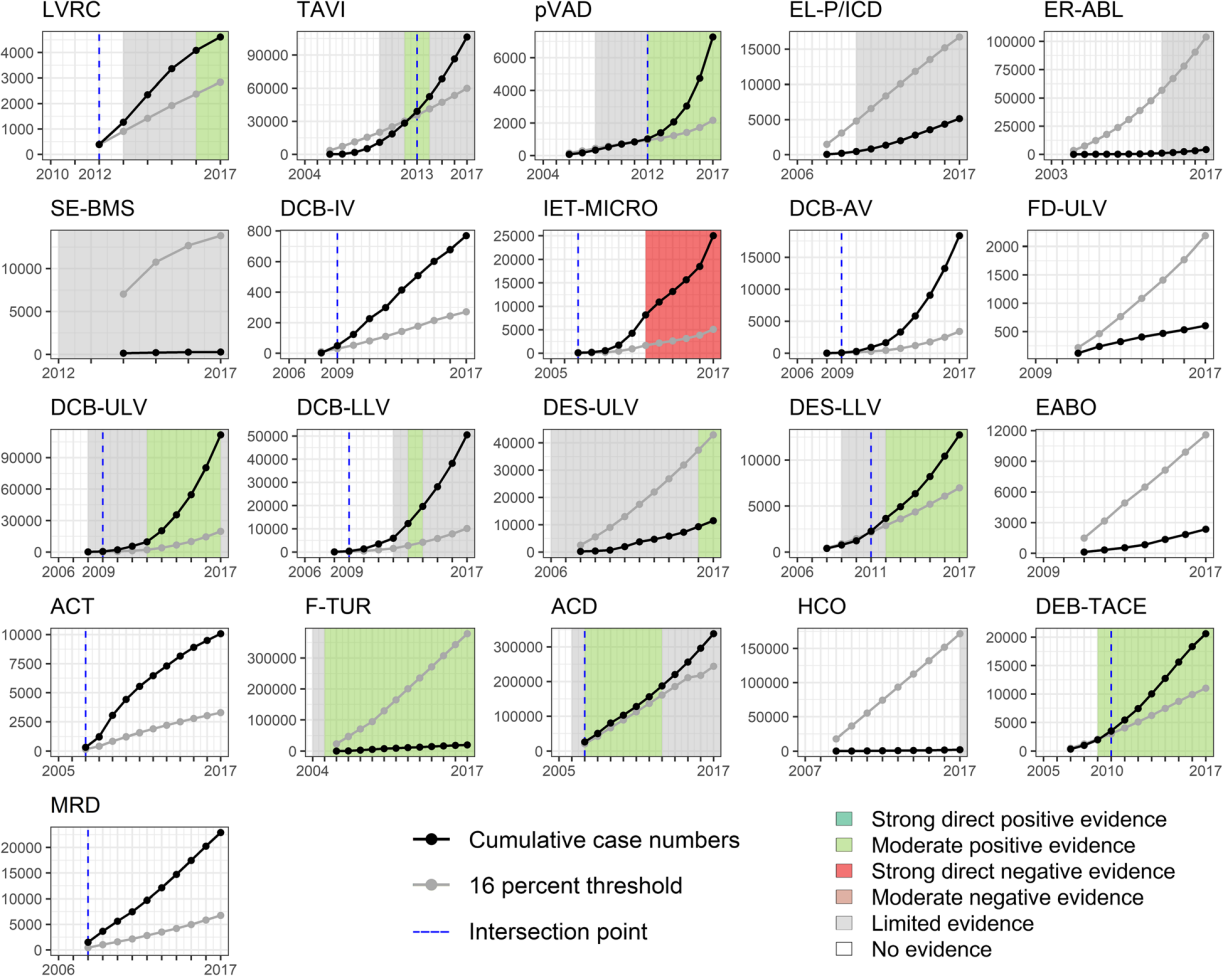
Diffusion curves and identified evidence

To determine the target population for each technology, we identified the predominant standard of care for each indication in the literature. We subsequently accessed the “DRG statistics” dataset remotely via the Research Data Center of the German Federal Statistical Office (see step 1 for information on the dataset). No definitive comparator or clear coding was available for 6 out of 27 technologies (PECLA/iLA, MVAC, BVS, DCB-CV, IABC, and FDT), so these were excluded from further analysis. For the remaining 21 technologies, we cumulated the case numbers to transform the adoption curves (see Additional file 1) into diffusion curves (see Fig. 4). The cut-off value was defined for each year as 16% of the sum of the case numbers of the comparator and the technology itself.

For each technology, diffusion patterns are evaluated per year based on the grid in Fig. 2. In the subsequent analysis, we view the adoption of innovative medical technologies as dynamic, allowing and accounting for change in status.

## Results

The sample of 21 technologies fully investigated in this article includes 14 technologies that are applied to the cardiovascular system: eleven of them are used in endovascular interventions in the management of acute or chronic degenerative cardiovascular diseases (TAVI, pVAD, SE-BMS, DCB-IV, IET-MICRO, DCB-AV, FD-ULV, DCB-ULV, DCB-LLV, DES-ULV, DES-LLV); two in the treatment of atrial fibrillation (EL-P/ICD, ER-ABL); one is used in surgery when extracorporeal circulation is required (EABO). Four technologies are applied to the urinary system, with a focus on surgical continence therapy (ACT), fluorescence-assisted transurethral resection (F-TUR), and dialysis (ACD, HCO). One technology (LVRC) concerns the respiratory system for the treatment of emphysema which may occur in a number of chronic lung diseases (e.g., COPD). Lastly, we examined drug-eluting beads for transarterial chemoembolization (DEB-TACE) and molecular monitoring of residual tumor burden (MRD).

Figure 4 shows the diffusion curves of the 21 technologies and the underlying evidence, as well as the threshold value and its intersection point with the diffusion curve. This defines the change from cautious to widespread utilization. Five technologies (LVRC, IET-MICRO, ACT, ACD, MRD) were already applied to more than 16% of the total population in the first documented year of use. Eight (EL-P/ICD, ER-ABL, SE-BMS, FD-ULV, DES-ULV, EABO, F-TUR, HCO) did not reach this threshold during the entire period of observation.

**Fig. 5:**
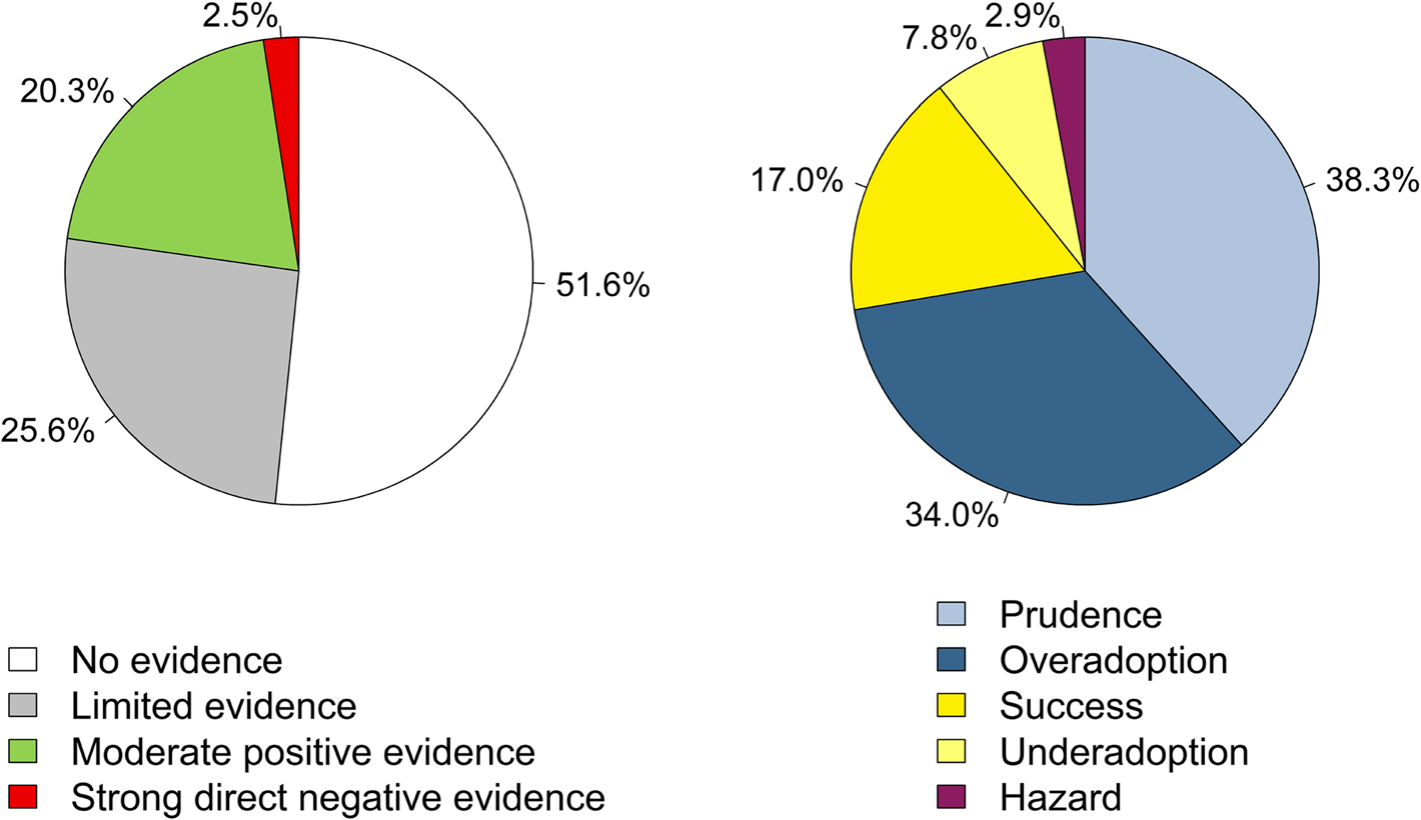
Distribution of evidence and diffusion patterns based on evaluated data years across considered technologies

**Fig. 6:**
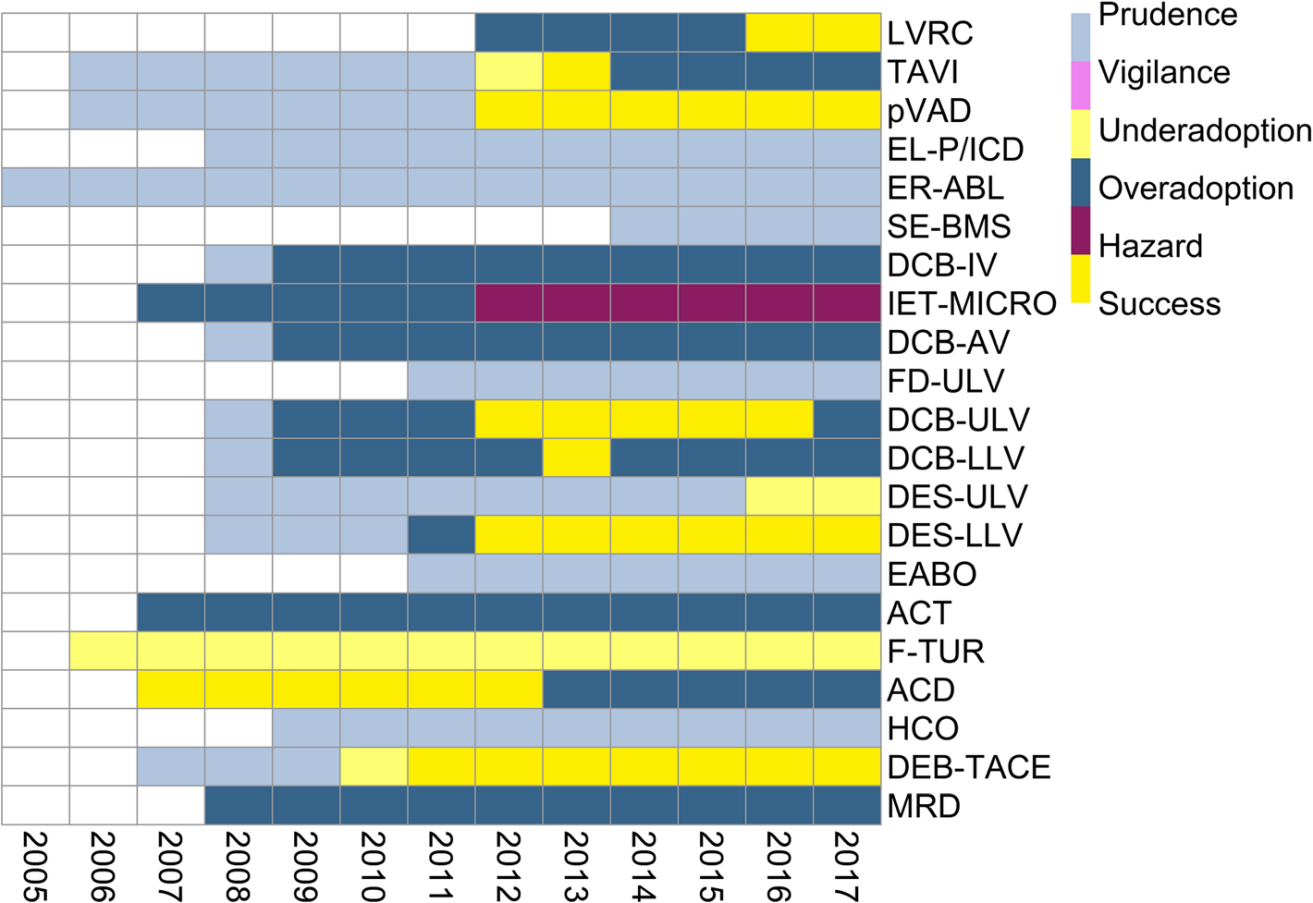
Heatmap of diffusion patterns

For the majority of technologies (16 of 21), no relevant study of acceptable scientific quality as defined for this work was available at the time of introduction. For more than half, no (6 of 21) or only limited (5 of 21) evidence was identified over the entire observation period. The evidence on four further technologies (TAVI, DCB-ULV, DCB-LLV, ACD) was considered “limited” at the end of the period because of conflicting study results. In contrast to the sparse evidence base, 13 out of 21 technologies were utilized widely over time. In relation to the years evaluated across all technologies, the proportion of no available (127 of 246 data years) or limited evidence (63 of 246 data years) predominates (see Fig. 5). Thus, the statuses “Prudence” (79 of 206) and “Overadoption” (70 of 206) together account for nearly three-quarters of the years evaluated, followed by “Success” with 17% (35 of 206). The “Vigilance” status (cautious utilization in light of negative evidence) was not assigned at all. From the perspective of technologies, successful diffusion was observed for eight out of 21 in at least 1 year (Fig. 6). Strong direct negative evidence was identified for only one technology (IET-MICRO), strong direct positive evidence for none. While moderate positive evidence was present in about 20% (50 of 246) of all data years, there were no data years with moderate negative evidence.

For the technology “anticoagulation with citrate during dialysis (ACD),” moderate positive evidence was already available at the time of its introduction into German inpatient care. Therefore, the process can initially be considered as “Success.” However, this changed over time to “Overadoption” due to inconsistent findings in several studies. Moderate positive evidence was also available for the procedure “fluorescence-assisted transurethral resection (F-TUR)” even 1 year before its introduction. However, throughout the entire observation period, the procedure was utilized cautiously in only a fraction of the target population.

**Fig. 7:**
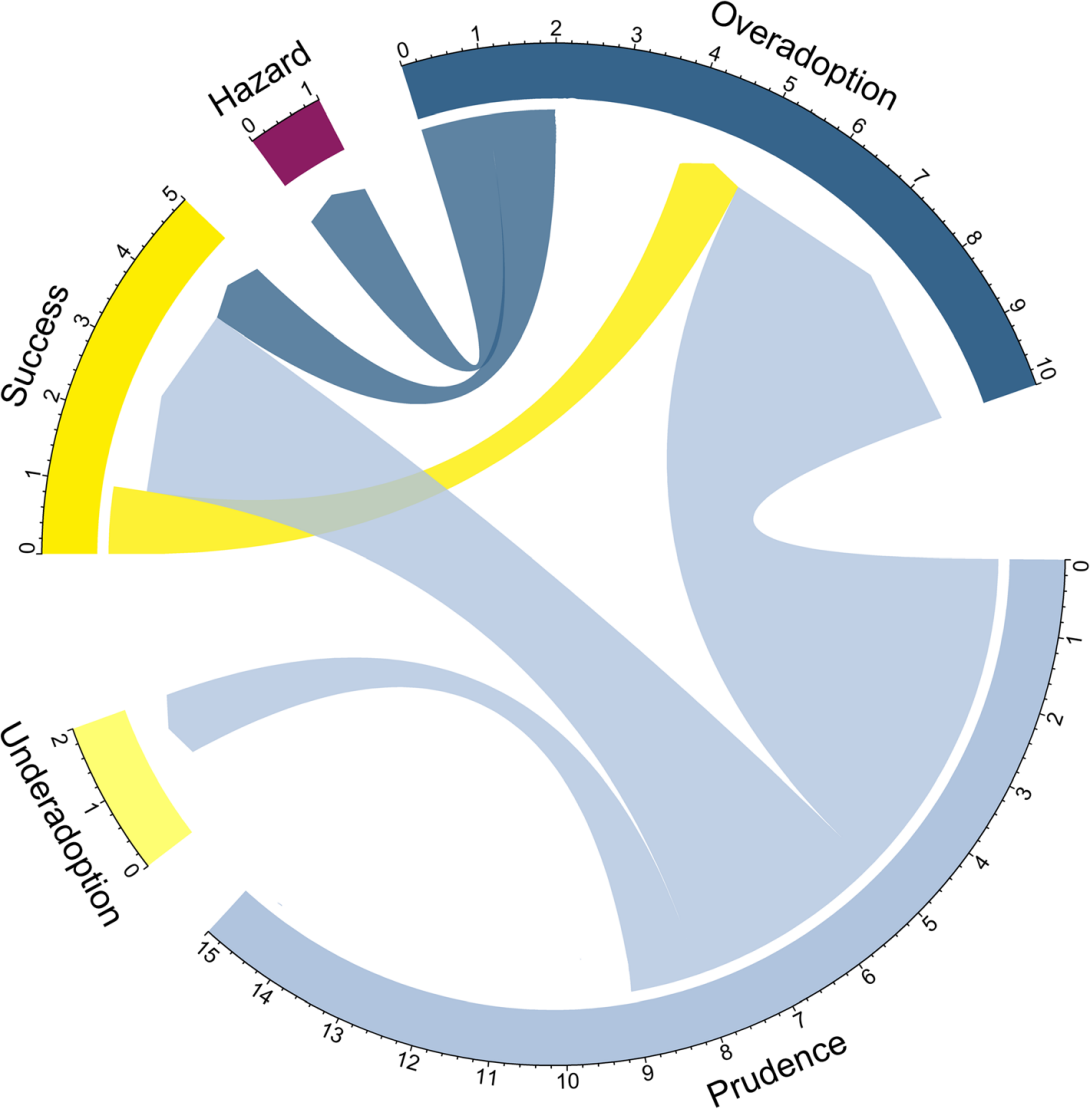
Status changes between start and end point of the observation period for considered technologies

Figure 7 visualizes the change in statuses between the start and end point of the observation period for all technologies based on the grid in Fig. 6. Only four technologies made a desirable shift from potential “Overadoption” (LVRC) or “Prudence” (pVAD, DES-LLV, DEB-TACE) to widespread utilization with supporting evidence (“Success”). Five technologies (TAVI, DCB-IV, DCB-AV, DCB-ULV, DCB-LLV) shifted from cautious to widespread utilization while either no evidence was identified or it became inconsistent over the observation period (“Overadoption”). On the contrary, the procedure “implantation of a drug-eluting stent in upper leg vessels (DES-ULV)” reached only a fraction of the target population although positive evidence prevailed at the end. Overall, adoption behavior changed from a focus on “Prudence” to “Overadoption.” While the statuses “Hazard” and “Underadoption” were each assigned one additional technology at the end of the observation period, “Success” acquired the second most, going from one at the start to four at the end (see Fig. 7).

All technologies together were applied to a total of 775,410, a mean of 36,924 and a median of 11,468 cases during the observation period. The group of technologies for which no credible or even negative evidence was available in 2017 was applied to an average of 46,565 cases (in total to 698,479 during the observation period; median 10,077), while those with positive study results were only applied to an average of 12,822 cases (in total to 76,931 during the observation period; median 12,101). The three technologies most relevant to care overall as determined by cumulative cases (ACD with 338,123 cases; DCB-ULV with 111,727 cases; and TAVI with 106,392 cases), all resulted in “Overadoption.”

## Discussion

This study aimed to explore the diffusion of new technologies in German inpatient care, focusing on the relationship between utilization and available scientific evidence. Previous research found a gap between existing rigorous evidence and lack of application of new technologies [20, 30, 31]. In the present study, however, we observe the opposite phenomenon. We found a generally sparse evidence base in terms of quantity and quality for the majority of technologies investigated. Thus, no or limited evidence could be found for 11 of the 21 technologies. Despite that, most investigated technologies (13 of 21) were utilized widely in German inpatient care. This entails a considerable risk for some technologies, particularly for invasive procedures such as the implantation of “self-expanding bare metal stents in coronary vessels (SE-BMS)” or “drug-coated balloon catheter in intracranial vessels (DCB-IV).” During the study’s observation period, German hospitals treated a total of about 700,000 cases with technologies without rigorous scientific support. However, the total body of evidence on a given technology is subject to rapid change. Accordingly, an initially positive evaluation may be followed by contradictory study results (see TAVI, DCB-ULV, DCB-LLV, ACD), e.g., if the target group of the technology is widened to include further population subgroups. Nevertheless, this does not imply that the benefit of the technology is negated overall.

In addition, study results must be disseminated from the moment of publication onward to reach potential adopters [32]; in our sample, user response to a change in evidence seems inhibited for some technologies. For ACD, after initial successful diffusion, the evidence base changed in 2013 due to an RCT showing negative effects of the technology. However, the adoption rate continues to steadily increase instead of declining (see Additional file 1). This can be explained in part by the momentum in diffusion of an innovation: The more hospitals adopt a technology, the more popular it becomes [16]. On the other hand, there are also positive examples (TAVI, pVAD, ACD, DEB-TACE) where the change to widespread utilization was in the same or only 1 year behind the turn in positive evidence. However, these findings also need to be considered cautiously. For instance, there is ongoing debate about the diffusion of “transcatheter aortic valve implantation (TAVI)” in Germany (where adoption rates are very high in the international comparison) in light of inconclusive evidence base [33]. In general, it should be noted that across examined technologies, most years evaluated are assigned to “Prudence.” According to Denis et al. [13], cautious utilization in the case of limited or lacking evidence can even be considered a success.

Concerning the change in diffusion patterns over time, we predominantly captured cases of users becoming more courageous in their use of technologies the benefits of which remain unclear. For example, the diffusion of “drug-coated balloon catheter in intracranial, abdominal, upper leg and lower leg vessels (DCB-IV, -AV, -ULV, -LLV)” was categorized as “Overadoption” in the end of the observation period. Regarding DCB-AV, the technology spreads rapidly from three cases in 2008 to a total of 18,318 cases in 2017, although no RCT at all could be identified. The evidence base for DCB-IV is similarly weak, with exceeding the threshold of widespread utilization like DCV-AV already in 2009, the year after introduction to the German health care system. Interestingly, the application of DCB in both ULV and LLV was intermediately considered as “Success.” The utilization rates increased above the threshold before findings of supportive evidence emerged, and adoption was therefore classified as “Overadoption” starting in 2009. However, in 2012, one RCT considering both application areas concluded that the technologies showed superior outcomes compared to standard balloon angioplasty [34]; this was corroborated by findings from another RCT in 2012 for DCB-ULV [35] and one in 2013 for DCB-LLV [36]. However, two RCTs with negative conclusions were published concerning the use in LLV in 2014 [37, 38], but the number of cases continued to increase. Therefore, a correlation between available study results and utilization rates cannot be assumed. For DCB-ULV, the conclusions of several RCTs were consistently positive until 2017 [39], so the impact of the negative findings remains to be seen.

We also observed an increase in technologies changing to successful diffusion in the end of the observation period. For example, the rapidly rising utilization rates of “implantation of a drug-eluting stent in lower leg vessels (DES-LLV)” were accompanied by supportive evidence. The first identified RCT was published in 2009 by Falkowsky et al. [40]. The authors classify the DES as an effective and safe technology that restores the original flow in the vessel and was associated with a lower risk of restenosis in comparison to bare metal stents. Already 2 years later, the annual case number was nearly three times higher and the utilization shifted from cautious to widespread. In 2012, primarily positive conclusions of three further RCTs were published indicating for instance a higher event-free survival rate and a reduced amputation rate [41–43]. In accordance, the annual case number of 2310 at the end of the observation period in 2017 is more than six times higher than in 2008.

A successful diffusion should maximize patient benefit and minimize potential risks. Ideally, the transfer of innovative medical technologies into practice should be guided and accompanied by the existing scientific evidence and induce the systematic generation of new data [44]. As only little or no evidence may be available at the time of market approval, evidence generation during the application of new technologies in routine care is necessary in order to obtain scientific knowledge [9]. However, the observed diffusion of innovative medical technologies in the German health care system fails to generate comprehensive and reliable evidence. The current reimbursement system for inpatient care aims to enable fast access to new medical technologies. According to our analyses, there is little need to further accelerate adoption processes as only 8% of the years analyzed show underadoption of technologies. However, this swift introduction of new medical technologies without proof of benefit should be accompanied by meaningful clinical studies to evaluate them as they are used [21]. This requires a high level of personnel and financial resources, effectively limiting the conducting of clinical trials to large (university) hospitals. In this context, it is the responsibility of policymakers to establish adequate yet not overly complex bureaucratic structures and targeted incentives that will enable appropriate hospitals to participate in evidence generation. At the same time, new knowledge must be made available to hospitals in a timely and systematized manner.

In addition to clinical studies, observational or “real-world” evidence can also be generated through medical device registries. Making the use of registries mandatory for innovative technologies could further enhance the evaluation of effectiveness and risks under routine practice conditions and provide insight into mid- and long-term outcomes. Thus, registries would offer valuable information for improving quality of care when incorporated into decision-making for (de)adopting new technologies [45]. What is more, linking clinical trials and registries (“registry-embedded clinical trials”) could accelerate the availability of robust evidence [46].

As mentioned, the level of reimbursement substantially influences the utilization of new technologies in German health care [21, 33]. Thus, the diffusion of innovative technologies could be managed by tying the reimbursement amount for technologies to the benefit they provide. Approaches such as “Coverage with Evidence Development (CED)” have already been implemented in many countries [47]. In the German health care system, the introduction of Article 137h Social Code Book V in 2016 established a pathway for the scientific evaluation of a technology’s benefit and harm as soon as hospitals request innovation payments for the first time [44]. However, only a fraction of all new medical technologies is affected by this regulation, specifically: “New diagnostic and treatment methods whose technical application is based essentially on a medical device of a high risk class with a particularly invasive character and a new theoretical-scientific concept” [48]. Therefore, the present CED approach in German inpatient care does not meet its goals due to lack of comprehensiveness and complexity [11, 47]. Also, in light of this study’s findings, there is potential for reconsideration.

Last but not least, successful diffusion also requires the de-implementation of low-value technologies that do not provide any benefit for patients, may have harmful consequences, and/or lead to a waste of health care resources [49–52]. Although international campaigns such as “Choosing Wisely” and “Too Much Medicine” emphasize the need of de-implementing interventions, there is little evidence on the most effective strategies to do so [53–55]. Nevertheless, the first step is the identification of low-value health care [54]. We observed lacking evidence for the majority of the technologies in our sample, which suggests that further efforts of systematic testing and generating evidence are necessary. To prioritize technologies for further evidence generation, criteria such as significant financial burden on health payers and the most expected value of information gained have been proposed [52]. In our study, these factors were incorporated as criteria for including technologies in the sample: we selected technologies that are more expensive than their alternatives (and require extrabudgetary payments) and affect a high volume of patients (demonstrated by relatively high utilization numbers). Further efforts are needed to develop clear de-implementation strategies including discussions on access to technologies, patient benefits, cost-benefit ratios, and innovation policy.

In brief, understanding technology diffusion requires an in-depth exploration of factors related to the technology itself, its potential adopters, and the context [56, 57]. The importance of considering elements such as previous experience of users and financial incentives has been established [19, 21, 58]. However, the (potential) influence of scientific evidence on the technology’s safety and effectiveness has for the most part only been explored theoretically or for isolated examples of technologies only. The main strengths of this study lie in its clear methodological approach to exploring this relationship empirically, and in a varied sample of 21 technologies. The identified gap between patterns of diffusion and evidence development reinforces the need for a renewed policy focus on how innovation systems are set up, concerning the prioritization and funding of necessary research on new technologies as well as embedding it in health care practice and linking it to (de)implementation decisions along the technology’s life cycle.

## Limitations

It is plausible that the following limitations may have influenced the results of this study. Despite our best efforts, it is possible that the systematic review missed relevant studies. Furthermore, the two-dimensional evaluation scheme does not take into consideration factors relevant to the diffusion of innovative medical technologies other than scientific evidence. The analysis is based on a sample obtained through defined criteria and an empirical selection process. Even though this was designed to yield a sample that was both varied and feasible, it is possible that a different sample would have resulted in different findings. Results for the 21 technologies investigated are not generalizable for all innovative medical technologies in an overarching way for several reasons. The final sample consists predominantly of cardiovascular interventions. Additionally, the initial sample of 59 technologies was based on the official list of technologies, for which hospitals applied for innovation payments. By definition, these lists do not include interventions that are less expensive than existing alternatives, but may still be invasive and potentially harmful. The selection criterion of overall relevance to care does not imply that non-selected technologies are irrelevant and should not be investigated or addressed. The sole focus on RCTs may have resulted in the loss of important results from other study designs. Systematic reviews and outcomes from observational studies can play an important role in clinical decision-making and may have influenced the implementation process [59, 60]. Dissemination bias may have skewed our results, as some results are neither published nor disseminated [32]. Lastly, both evidence generation and diffusion are dynamic, so the present analysis constitutes a snapshot illustrative of the observation period only.

## Conclusions

This study makes a substantial contribution to research on the diffusion of innovative medical technologies in German inpatient care. We analyzed diffusion patterns and evidence development for 21 technologies using quantitative and qualitative methods. We observed that the diffusion rate of new technologies is often not in line with available scientific evidence. At the end of the observation period, the share of overadopted technologies predominated, which indicates considerable potential for harm to patients and inefficiencies for the health care system. Overall, there is a lack of evidence regarding the efficacy/effectiveness and benefit of innovative medical technologies utilized in German inpatient care. Even in cases of available evidence, the transfer of knowledge into practice seems inhibited. The successful diffusion of innovation requires substantial further efforts by policymakers to strengthen systematic knowledge generation and translation. Responsive policies must facilitate the diffusion of valuable technologies in health care and curb the spread of potentially dangerous and ineffective technologies. On the one hand, creating an environment that encourages the conduct of rigorous studies, promotes knowledge translation, and rewards innovative medical technologies according to their added value is a prerequisite for the diffusion of “true” innovations in the health care sector. On the other hand, supporting the development of de-implementation strategies is crucial, not least for the German health care system.

## Supplementary Information

**Additional file 1:** Adoption curves for all 59 technologies.

**Additional file 2:** Additional description of the methodology.

## Data Availability

The datasets analyzed during the current study are available from the corresponding author on reasonable request.

## Abbreviations

ACCS: Antibody coated coronary stent
ACD: Anticoagulation with citrate during dialysis
ACT: Adjustable continence therapy
BRA: Baroreceptor activation
BS-PV: Insertion of coated (covered) stents with bioactive surface for peripheral vessels
BS-VSAV: Insertion of coated (covered) stents with bioactive surface for visceral and supraaortic vessels
BVS: Bioresorbable vascular scaffold in coronary vessels
CBS: Coronary bifurcation stents
CE: Conformité Européenne
DCB-ARTV: Drug-coated balloon catheter in artificial vessels
DCB-AV: Drug-coated balloon catheter in abdominal vessels
DCB-CV: Drug-coated balloon catheter in coronary vessels
DCB-IV: Drug-coated balloon catheter in intracranial vessels
DCB-LAV: Drug-coated balloon catheter in lower arm vessels
DCB-LLV: Drug-coated balloon catheter in lower leg vessels
DCB-OTHV: Drug-coated balloon catheter in other vessels
DCB-SUAV: Drug-coated balloon catheter in shoulder and upper arm vessels
DCB-TV: Drug-coated balloon catheter in thoracic vessels
DCB-ULV: Drug-coated balloon catheter in upper leg vessels
DCB-VV: Drug-coated balloon catheter in visceral vessels
DEB-TACE: Drug-eluting beads for transarterial chemoembolization
DES-LLV: Implantation of a drug-eluting stent in lower leg vessels
DES-SAA: Drug-eluting stents for the treatment of lesions of the supraaortic arteries
DES-ULV: Implantation of a drug-eluting stent in upper leg vessels
EABO: Endoaortic balloon occlusion with extracorporeal circulation
EBV: Endobronchial valve
EL-P/ICD: Excimer laser extraction of pacemaker and defibrillator electrodes
ER-ABL: Cardiac event recorder after ablative measures for atrial fibrillation/atrial tachycardia
EU: European Union
EVCT: Ex vivo chemosensitivity testing
FDA: Food and Drug Administration
FD-IV: Flow-diverter (hemodynamically effective implant for endovascular treatment) in intracranial vessels
FDT: Fetoscopic drainage therapy
FD-ULV: Flow-diverter (hemodynamically effective implant for endovascular treatment of peripheral aneurysms) in upper leg vessels
FE-AAA: Fenestrated endoprostheses for abdominal aortic aneurysms
F-TUR: Fluorescence-assisted transurethral resection
G-BA: Federal Joint Committee
G-DRG: German Diagnosis Related Groups
HCI: Hybrid cochlear implant
HCO: Dialysis with high cut-off dialysis membrane
IABC: Bioactive coils for intracranial aneurysm therapy
IABC-EL: Bioactive extra-long coils for intracranial aneurysm therapy
IAELC: Extra-long coils (3D) for intracranial aneurysm therapy
IAHEI: Intraaneurysmal hemodynamically effective implant for endovascular treatment of intracranial aneurysms
IAVC: Volume coils for intracranial aneurysm therapy
IET-MICRO: Intracranial endovascular thrombectomy (microwire retriever)
InEK: German Institute of the Hospital Remuneration System
LVRC: Lung volume reduction by insertion of coils
MDS: Medical Review Board of the Federal Association of Sickness Funds
MESI: Esophageal sphincter implant, magnetic
MRD: Molecular monitoring of residual tumor burden
MR-PTC: Percutaneous transluminal clipping for mitral valve regurgitation
MVAC: Mitral valve annuloplasty with clamp
MVR: Minimally invasive operations on heart valves (implantation of a mitral valve replacement)
NEURO: Neurostimulator for stimulation of the spinal cord or peripheral nervous system, rechargeable
PECLA/iLA: Pumpless extracorporal lung assist/interventional lung assist
pVAD: Percutaneous ventricular assist device (microaxial blood pump)
PVR: Minimally invasive heart valve surgery (endovascular implantation of pulmonary valve replacement)
RCT: Randomized controlled trial
SCO-MAGN: Therapy of scoliosis by means of magnetic-controlled rods
SE-BMS: Self-expanding bare metal stents in coronary vessels
SE-DES: Coronary stent, self-expanding (at least two stents, drug-eluting)
S-ICD: Defibrillator with subcutaneously implantable electrode
SP-ENDOST: Endovascular implantation/repair of a stent prosthesis using an endostapler
TAVI: Transcatheter aortic valve implantation
UD-DJMS: Double J metal stent for urinary diversion in ureteral strictures
VEPTR: Vertical expandable prosthetic titanium rib
