# Correction to: Implementation of innovative medical technologies in German inpatient care: patterns of utilization and evidence development

**DOI:** 10.1186/s13012-022-01187-7

**Published:** 2022-01-24

**Authors:** Marie Dreger, Helene Eckhardt, Susanne Felgner, Hanna Ermann, Hendrikje Lantzsch, Tanja Rombey, Reinhard Busse, Cornelia Henschke, Dimitra Panteli

**Affiliations:** 1 Department of Health Care Management, Technische Universität Berlin, Straße des 17. Juni 135, 10623 Berlin, Germany; 2 Berlin Centre for Health Economics Research (BerlinHECOR), Technische Universität Berlin, Straße des 17. Juni 135, 10623 Berlin, Germany

**Correction to: Implementation Sci 16, 94 (2021)**

**https://doi.org/10.1186/s13012-021-01159-3**

Following publication of the original article [1], it was reported that Hanna Ermann’s name was erroneously given as ‘Hanna Errmann’.

The original article has been updated.

## References

[1] Dreger M, Eckhardt H, Felgner S (2021). Implementation of innovative medical technologies in German inpatient care: patterns of utilization and evidence development. Implementation Sci.

