# Supplementary material for: Implementation of innovative medical technologies in German inpatient care: patterns of utilization and evidence development
Source: Implement Sci. 2021 Oct 30;16:94. doi: 10.1186/s13012-021-01159-3 (PMC8556925; doi:10.1186/s13012-021-01159-3)
Supplement: Supplementary file 1 — Additional file 1: Adoption curves for all 59 technologies. [file 13012_2021_1159_MOESM1_ESM.pdf]

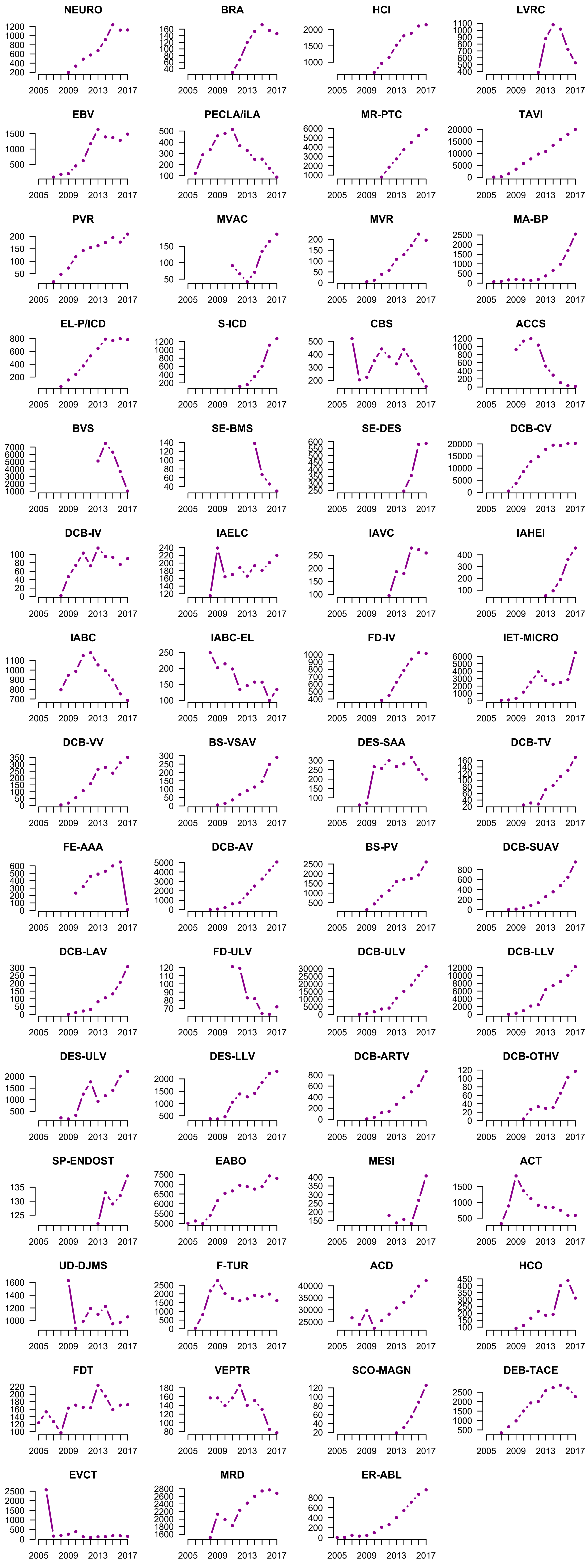
NEURO
BRA
HCI
LVRC
EBV
PECLA/iLA
MR-PTC
TAVI
PVR
MVAC
MVR
MA-BP
EL-P/ICD
S-ICD
CBS
ACCS
BVS
SE-BMS
SE-DES
DCB-CV
DCB-IV
IAELC
IAVC
IAHEI
IABC
IABC-EL
FD-IV
IET-MICRO
DCB-VV
BS-VSAV
DES-SAA
DCB-TV
FE-AAA
DCB-AV
BS-PV
DCB-SUAV
DCB-LAV
FD-ULV
DCB-ULV
DCB-LLV
DES-ULV
DES-LLV
DCB-ARTV
DCB-OTHV
SP-ENDOST
EABO
MESI
ACT
UD-DJMS
F-TUR
ACD
HCO
FDT
VEPTR
SCO-MAGN
DEB-TACE
EVCT
MRD
ER-ABL
2005
2009
2013
2017
