# Supplementary material for: Implementation of innovative medical technologies in German inpatient care: patterns of utilization and evidence development
Source: Implement Sci. 2021 Oct 30;16:94. doi: 10.1186/s13012-021-01159-3 (PMC8556925; doi:10.1186/s13012-021-01159-3)
Supplement: Supplementary file 2 — Additional file 2: Additional description of the methodology. [file 13012_2021_1159_MOESM2_ESM.docx]

**Additional file 2: Additional description of the methodology**

The requirements for performing a cluster analysis were implemented as follows. First, the use of comparative measures requires that the data to be analyzed are in a uniform and, in this specific case, metric scale level. However, missing values occurred naturally in the data set, as technologies were introduced in inpatient care at different points of time and case numbers were therefore not consistently available for all years in the observation period. Missing values were assigned the abbreviation for not available (NA). This expression is interpreted by the statistical software R as different from zero. To ensure that the grouping of technologies was independent from the different introduction times, all curve progressions along the x-axis were normalized to a common starting point. It was assumed that the development of case numbers for each innovation does not depend on the respective year, but on the past years since the introduction of the technology. Furthermore, the case numbers $f$ of each technology $i$ of the years $t_{2}$ to $t_{13}$ were normalized to the starting value $f_{i}\left( t_{1} \right)$ to ensure that the clustering was based on the development of the diffusion curve and not on the absolute level of case numbers, which strongly depend on indication and purpose of the technology.

Since the number of clusters was not set a priori, a hierarchical agglomerative clustering method was applied. Starting with single technologies, clustering was performed across several small and homogeneous overlap-free aggregates until an all-encompassing aggregate was reached. The homogeneity of these aggregates decreased with their size, up to the maximum heterogeneity when the initial sample was reached [1]. Due to its performance strength when applied to metric data, the analysis in the present case was performed using Ward's algorithm based on the Euclidean distance between two curves as a proximity measure [1, 2]. The optimal number of groups that are as homogeneous as possible was determined using the so-called elbow criterion. For this purpose, the growing homogeneity was plotted against the number of clusters (see Figure 2-1).


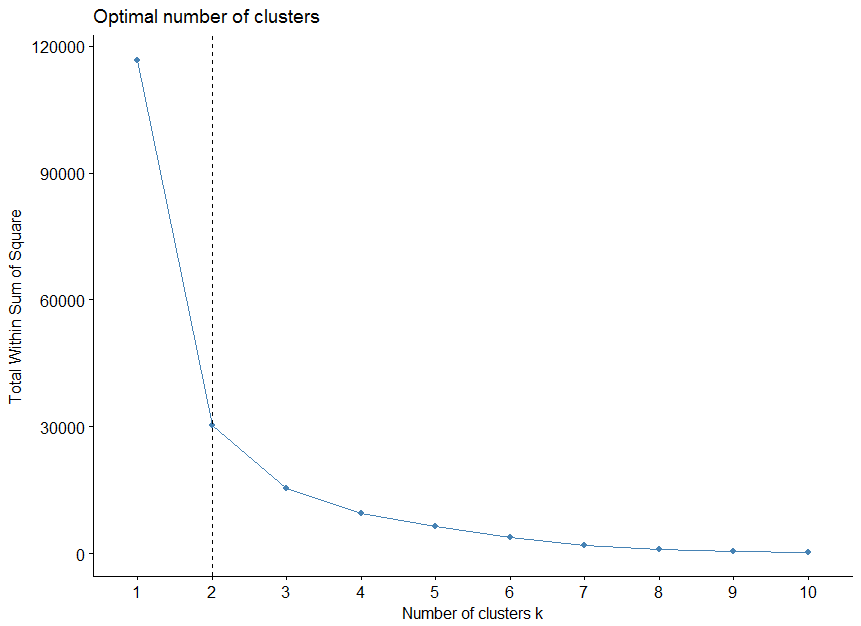


Figure 2-1: Defining the optimal number of clusters according to the elbow criterion

The "elbow" in the curve thus leads to a result of two clusters [3]. The assignment of the technologies to clusters could then be determined from the dendrogram (see Figure 2-2). This shows the hierarchical agglomeration from the single-element groups to an entire aggregate of the sample.


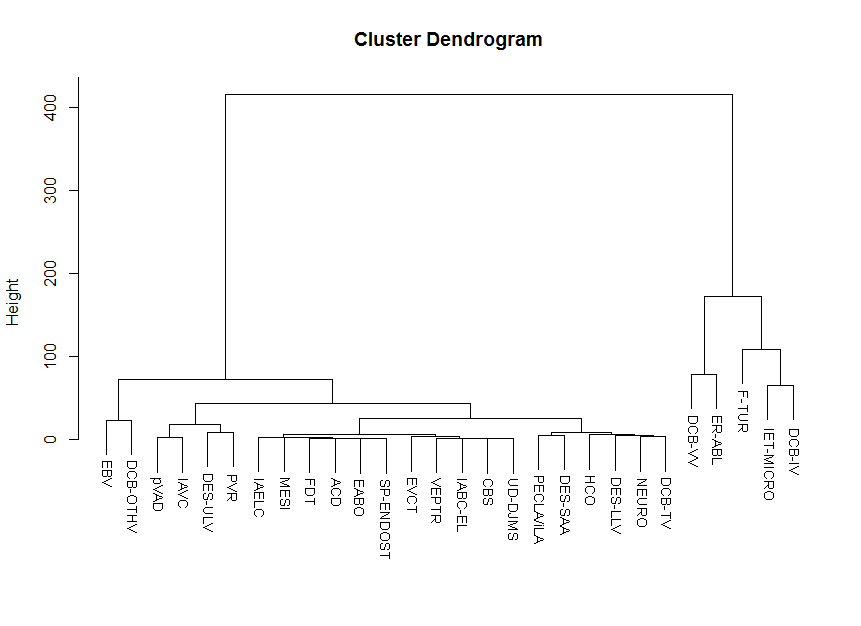


Figure 2-2: Cluster Dendrogram

References

1. Stein P, Vollnhals S. Grundlagen clusteranalytischer Verfahren: Institut für Soziologie - Universität Duisburg-Essen; 2011.

2. Murtagh F, Legendre P. Ward’s Hierarchical Agglomerative Clustering Method: Which Algorithms Implement Ward’s Criterion? J Classif. 2014;31:274–95. doi:10.1007/s00357-014-9161-z.

3. Wolf C, Best H. Handbuch der sozialwissenschaftlichen Datenanalyse. Wiesbaden: VS Verlag für Sozialwissenschaften; 2010.
